# A systematic review evaluating the efficacy of autologous hematopoietic transplantation for diffuse large B cell lymphoma-type Richter syndrome

**DOI:** 10.22088/cjim.14.1.1

**Published:** 2023

**Authors:** Mehran Sharifi, Ziba Farajzadegan, Saeid Rezaei Jouzdani

**Affiliations:** 1 Department of Internal Medicine, School of Medicine, Cancer Prevention Research Center, Seyyed Al-Shohada Hospital, Isfahan University of Medical Sciences, Isfahan, Iran; 2 Department of Community and Preventive Medicine, Faculty of Medicine, Isfahan University of Medical Sciences, Isfahan, Iran

**Keywords:** Richter, Autologous, Hematopoietic transplantation, Efficacy, Systematic review

## Abstract

**Background:**

Chronic lymphocytic leukemia (CLL) can transform into fast growing lymphoma for diffuse large B-cell lymphoma (DLBCL) called Richter's syndrome (RS), which is commonly related to an existence of large B-cells with equal or larger size than macrophage nuclei or more than twice those of normal lymphocyte. We conducted a systematic review of the existing literature to assess the clinical efficacy of auto-HCT for patients with RS.

**Methods:**

We searched 4 main databases; EMBASE, Google Scholar, Scopus, PubMed and Web of Science and was done on December 26, 2021. All analyses in this study were performed by Stata software and this review was reported in accordance with PRISMA 2020.

**Results:**

Data was extracted from 4 articles; the total number of patients was reported to be 110. Based on the meta-analysis findings, pooled overall survival rate was 56.36% (95%CI= (46.98–65.31). In figure 2, the forest plot of combined results is shown.

**Conclusion:**

Despite the use of common treatment regimens such as chemo immunotherapy and the availability of novel therapies including B-cell receptor inhibitors and rituximab-cyclophosphamide-hydroxydaunorubicin-Oncovin-prednisone (CHOP-R) regimen, the status of disease progression and recovery in RS cases is still not strong enough.

Chronic lymphocytic leukemia (CLL) can transform into fast growing lymphoma for diffuse large B-cell lymphoma (DLBCL) called Richter's syndrome (RS), which is commonly related to an existence of large B-cells with equal or larger size than macrophage nuclei or more than twice those of normal lymphocyte. This syndrome occurs in nearly 2 to 10% of CLL patients during their illness duration with associated poor clinical outcome (1, 2). Evidence has shown that multi-agent immune-chemotherapies could bring a relatively protective response among RS patients with an overall response rate of 40% and overall survival duration of less than a year (3, 4). Related poor outcomes might be explained by a unique and more complex genomic landscape in RS patients than those with chronic lymphocytic leukemia without RS (5, 6). Consequently, the limited efficacy of traditional therapies and the emergence of new therapeutics in patients with RS, resulted in the announcement of American Society for Blood and Marrow Transplantation proposing that a hematopoietic cell transplantation (HCT) to patients after an objective response to chemotherapy could be clinically beneficial (7-9). 

Furthermore, as the response duration with chemotherapy is short, both autologous and allogeneic stem cell transplantation have been suggested as consolidation chemotherapy to produce durable remission in patients with DLBCL-type RS (10). A stem cell transplant is a process in which physicians replace a patient's diseased stem cells with new, healthy ones. This process has been proven to be effective and lifesaving for *people with blood cancers* and *disorders, including leukemia, lymphoma* and myeloma (11). In autologous hematopoietic stem-cell transplantation (Auto-SCT), an intrinsically normal patient's own stem cells are collected to allow blood cell recovery after undergoing high dose therapy. Afterward, healthy stem cells are replaced in the body to produce white blood cells, red blood cells and platelets (12). On the other hand, allogeneic hematopoietic stem-cell transplantation (Allo-SCT) is a procedure in which a patient receives healthy stem cells from the blood or bone marrow of a donor to restore their own stem cells (11). 

Although the European Group for Blood and Marrow Transplantation (EBMT) has approved the efficacy of both Auto-HCT and allo-SCT in DLBCL-type RS, most of the patients cannot achieve an optimal response to go on to stem cell transplant (10). Respectively, findings of a study conducted by Cwynarski et al. revealed that only a minority group of these patients may benefit from stem cell transplantation. In this study, during a three-year follow-up of patients, relapse-free survival was reported to be 27% after allo-HCT and 45% after Auto-HCT. Furthermore, the non-relapse mortality during this period of time was 26% after allo-HCT and 12% after auto-HCT. Finally, the survival rate after mentioned therapies was respectively reported to be 36% and 59% (13). 

Despite significant advances in the field, research on the use of auto-SCT or allo-SCT in the treatment of RS is limited (14). Moreover, to our knowledge, there are no systematic review and meta-analysis that investigate overall survival, relapse, partial response rate, complete remission rate, and non-relapse mortality following an auto-SCT. Therefore, we conducted a systematic review of the existing literature to assess the clinical efficacy of auto-HCT for patients with RS.

## Methods

**Databases and search terms:** A systematic search of EMBASE, Google Scholar, Scopus, PubMed and Web of Science was done on the 26^th^ of December 2021 (Table 1). 

**Table 1:** Search Strategy based on databases

**Database**	**Search Strategy**
PubMed	((“Richter *”[ TITLE-ABSTR-KEY]) AND (“Hematopoietic Stem Cell Transplantation *”[TITLE-ABSTR-KEY] ) AND (“Diffuse Large B-Cell Lymphoma*”[TITLE-ABSTR-KEY] ))
Web of Science	TS= (“Richter *”) AND TS= (“Hematopoietic Stem Cell Transplantation *”) AND TS= (“Diffuse Large B-Cell Lymphoma*”)
Embase	'Richter*':ab,ti AND 'Hematopoietic Stem Cell Transplantation *:ab,ti AND 'Diffuse Large B-Cell Lymphoma *:ab,ti
Scopus	TITLE-ABS-KEY (Richter*) AND TITLE-ABS-KEY ('Hematopoietic Stem Cell Transplantation* ) AND TITLE-ABS-KEY ('Diffuse Large B-Cell Lymphoma* )
Google Scholar	“Richter*” AND “Hematopoietic Stem Cell Transplantation *” AND “'Diffuse Large B-Cell Lymphoma*” anywhere in papers

The search terms included ((((Richter syndrome) OR (Richter)) OR (richter)) AND (((((Hematopoietic Stem Cell Transplantation[Title/Abstract]) OR (Stem Cell Transplantations [Title/Abstract])) OR (Stem Cell Transplantation[Title/Abstract])) OR (Transplantations [Title/Abstract])) OR (Transplantation[Title/Abstract]))) AND ((((((((((((B-Cell Lymphoma[Title/Abstract]) OR (B Cell Lymphoma [Title/Abstract])) OR (B-Cell Lymphomas [Title/Abstract])) OR (Histiocytic Lymphomas [Title/Abstract])) OR (Diffuse Large-Cell Lymphoma [Title/Abstract])) OR (Diffuse Large Cell Lymphoma [Title/Abstract])) OR (Diffuse Large-Cell Lymphomas [Title/Abstract])) OR (Histiocytic Lymphoma [Title/Abstract])) OR (Diffuse Histiocytic Lymphoma [Title/Abstract])) OR (Diffuse Histiocytic Lymphomas [Title/Abstract])) OR (Diffuse Large B-Cell Lymphoma [Title/Abstract])) OR (Diffuse Large B Cell Lymphoma [Title/Abstract])). In an initial search of electronic databases, 431 records were identified. An additional search in Google Scholar also resulted in 7 articles. After importing the records to EndNote software and removing the duplicates, 283 articles remained to be screened through the review of title/ abstracts by two research team members. Studies which incorporated quantitative data on the efficacy of autologous hematopoietic transplantation for diffuse large B cell lymphoma-type Richter syndrome. Conference abstracts and references of included articles were also searched to find any relevant data to be added in the review. 

**Inclusion and exclusion criteria:** Descriptive, prospective, cross-sectional, case-control, case-series, randomized controlled trials and cohort studies published in English in December, 2021 to determine the efficacy of autologous hematopoietic transplantation for diffuse large B cell lymphoma-type Richter syndrome. Also, studies must have enrolled patients who ended up receiving ASCT for the sole purpose of DLBCL type Richter syndrome. Selection of included studies was undertaken by two authors. Possible disagreements were resolved by consensus majority of authors. Papers with the study designs of review, brief reports, and letter to the editor, expert opinions, editorials, book chapters, commentaries, thesis were not included in the research. Furthermore, the articles in languages other than English, or published after February 2021 were excluded. Also, we did not apply any search limits based on language, country of origin and studies that were reported only in abstract form.

**Figure 1:**
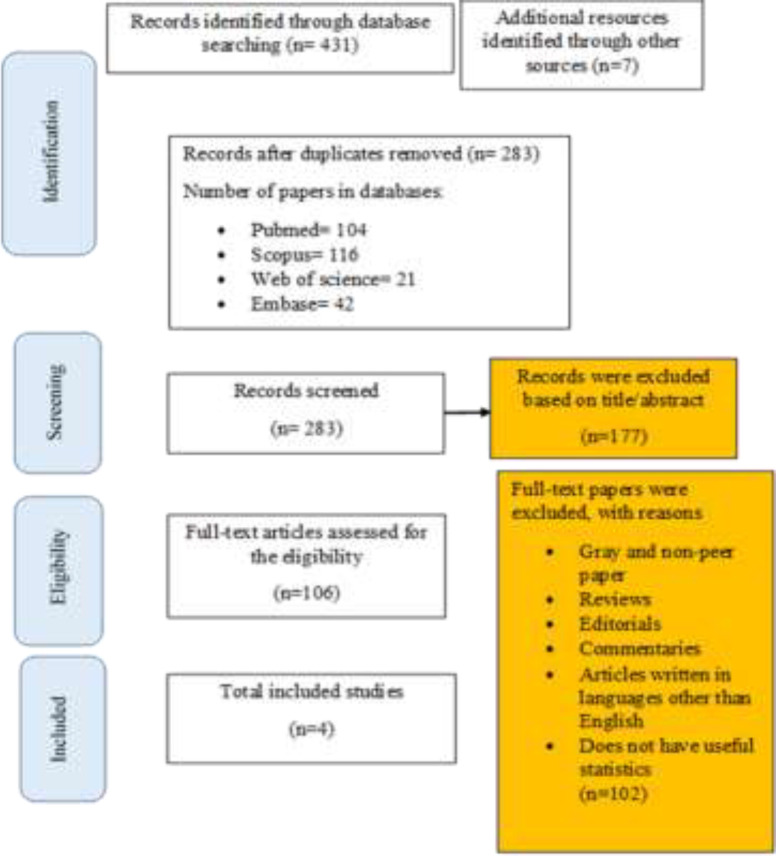
Flow diagram of our review process (PRISMA)

**Selection process:** In the first step of search process, 438 records were found. After removing duplicates, 283 articles remained; of which 104 articles were published in PubMed, 116 in SCOPUS, 21 in Web of Science and 42 articles were retrieved from EMBASE. The screening of title/ abstracts led to 106 relevant records. In the final step, after considering the inclusion/ exclusion criteria, 4 articles were included in the review (13, 15-17) (figure 1). 

**Data extraction:** Two investigators extracted the study data independently through the use of a data extraction form encompassing name of author/ authors, date of publication, research setting, study design and also clinical outcome data extracted from eligible studies in relation to benefit and harms. To assess clinical benefits, we extracted data on overall response rate (ORR), complete remission, overall survival (OS), progression free survival (PFS). For harm data, extract non relapse mortality (NRM), relapse after ASCT were extracted (Table 2).

**Quality assessment:** Newcastle-Ottawa Scale (NOS) was used to assess the quality of included articles. To minimize bias, two independent reviewers evaluated the quality of studies and in case of any disagreement, a third investigator resolved the discrepancy. The NOS uses a star system to judge the quality of included articles based on three perspectives including the ascertainment of either the exposure or outcome of interest for related types of study, selection of study groups, and their comparability. This scoring includes three points for ascertainment of exposure and outcomes, four points for selection of study groups, and two points for their comparability. A maximum of 9 points can be assigned to each article with studies having a total score of ≥7 defined as high quality and a score below 4 was considered as a low quality article (18).

**Statistical Analysis:** In this systematic review, we assess heterogeneity among the studies using the I^2^ test. Moderate heterogeneity is defining as I^2^>30%, and high heterogeneity as I^2^>60%. We report all results as rates with their corresponding 95% confidence intervals (CIs). We perform all analysis in this study via Stata software and this review is reported in accordance with PRISMA 2020 (Preferred Reporting Items for Systematic Reviews and Meta-Analyses) checklist (19).

**Table 2:** Characteristics of included studies

Study	Year	Study type	Study period	Patients With Auto SCT	n (Male sex)	Median Age (years)	Median follow up months	PR=partial response	CR = complete remission	OS = overall survival	NRM = non-relapse mortality	Relapse	progressi-free survival	relapse free survival	Regimenintensity	Quality of Study
**Cwynarski et al. (** 13 **)**	2012	Registry (EBMT)	1997 to 2007	34	23	57(31–70)	30 (11 to 90)	50%	32%	59% CR,Cru,PR	12%	43.00%	NG	45%	MAC = NA RIC = NA	High
**Herrera et al. (** 15 **)**	2021	Observational studies(CIBMT)	2007 to 2017	53	37	65 (30-79)	48 (4 to 98)	28%	66%	57% CR, PR Stable/untreated	15%	33.33%	48%	NG	MAC =NA RIC = NA	High
**Tsimberidou et al. (** 16 **)**	2006	Single institution	1975 to 2005	3	2	60 (35–72)	NE	NG	NG	33.33% CR, CRu, PR,	NG	NG	NG	NG	MAC = 3 RIC = 0	High
**Wang et al. (** 17 **)**	2020	Single institution	1993 to 2018	20	12	62 ( 41-73)	67	NG	NG	55%	NG	NG	NG	NG	MAC = NA RIC = NA	High

## Results

Our findings are reported based on the PRISMA. Following the extraction of data from 4 articles, the total number of patients was reported to be 110 (figure 1).

**Overall survival: **Four studies reported the overall survival in RS patients who underwent auto-SCT Richter. In these researches, 110 patients with an average age of 61 years old were examined.

**Figure 2:**
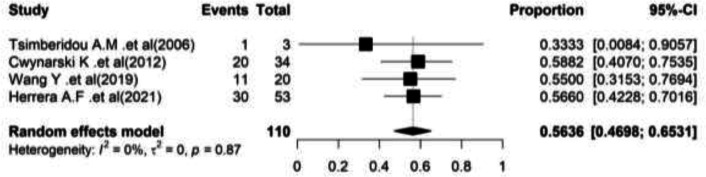
Forest plot of Overall survival

 Most of the cases (67.27%) were males. The heterogeneity between studies was not significant (I^2^=0.00%, Q-value=0.70, df=3, P=0.87). Based on the meta-analysis findings, pooled overall survival rate was 56.36% (95%CI=(46.98–65.31). In figure 2, the forest plot of combined results is shown (figure 2).

**Relapse: **In two studies, relapse and partial response rates were reported in patients with Richter’s syndrome who underwent autologous stem cell transplantation. In these researches, 87 patients with an average age of 61 years old and the majority of male gender (54.54%) were investigated. The heterogeneity between studies which analyzed the relapse rate was not significant (I^2^=0.00, Q-value=0.35, df=1, P=0.55), while the heterogeneity between researches conducted to analyze partial response rate was significant (I^2^=75.6, Q-value=4.10, df=1, P=0.042).

**Figure 3:**
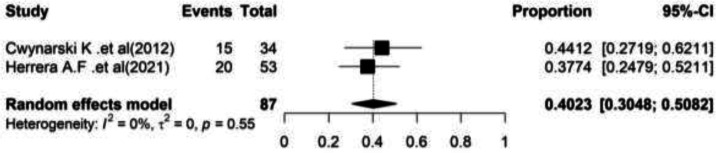
Forest plot of Relapse

 Based on the meta-analysis findings, pooled relapse rate was reported at 40.23% (95%CI= (30.48 – 50.82) (figure 3).

**Figure 4:**
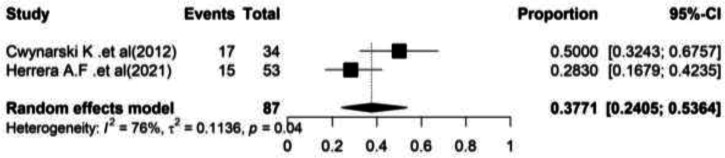
Forest plot of Partial Response (PR)

**Partial Response (PR): **In figure 4, the forest plot of combined results is shown. Based on the meta-analysis, the pooled partial response rate in this group of patients was 37.71% (95%CI= (24.05–53.64) and the heterogeneity between studies was significant (I^2^=75.6, Q-value=4.10, df=1, P=0.042) (figure 4).

**Figure 5:**
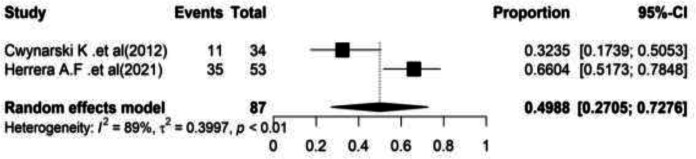
Forest plot of Complete Remission (CR)

**Complete Remission (CR): **Furthermore in two studies, the meta-analysis results reported the pooled complete remission at 49.88% (95%CI= (27.05–72.76) with a significant heterogeneity (I2=88.9, Q-value=9.00, df=1, P=0.002) (figure 5).

**Figure 6:**
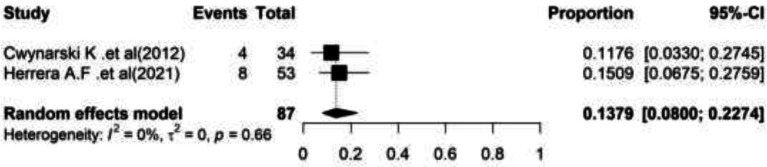
Forest plot of Non-Relapse Mortality (NRM)

**Non-Relapse Mortality (NRM): **The non-relapse mortality based on the meta-analysis of two studies was estimated at 13.79% (95%CI=(8.00–22.74) with a significant heterogeneity (I2=0.00, Q-value=0.19, df=1,P=0.66) (figure 6). 

**Publication Bias: **According to the analysis regarding the detection of publication bias, there was no publication bias (t-value=1.17, df=2, P=0.36). 

**Figure 7:**
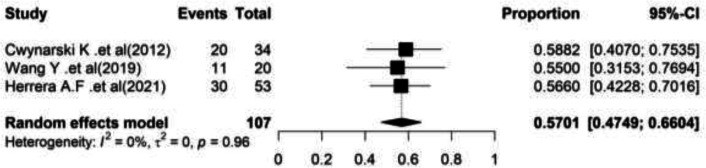
Forest plot of Sensitivity analysis

**Sensitivity analysis: **One of the studies with less than 3 samples who underwent auto-SCT, to control the effect of small sample size on pooled overall survival, the mentioned study was discarded from the meta-analysis (figure 7).

## Discussion

Despite the use of common treatment regimens such as chemo immunotherapy and the availability of novel therapies including B-cell receptor inhibitors and rituximab-cyclophosphamide- hydroxydaunorubicin- Oncovin prednisone (CHOP-R) regimen, the status of disease progression and recovery in RS cases is still not strong enough (14). In addition, literature has shown that in such cases, novel therapeutic approaches such as ibrutinib could not lead to long-lasting responses (20, 21). The reported limitations in the efficacy of available therapies highlight the necessity for better recognition of potential clinical value of stem cell transplantation for the treatment of patients with Richter's syndrome. Thus, we performed a systematic review and meta-analysis to summarize the quantitative data of the published literature about the evaluation of the outcomes after autologous stem-cell transplantation (13). 

Findings of this review revealed that auto-SCT resulted in the overall survival of 56.36% with a pooled relapse rate of 40.23% indicating that using novel therapies including auto-HCT can increase the survival rate and reduce the risk of relapse to ultimately improve the clinical outcomes in patients. With reference to the pooled non-relapse mortality (NRM) of 13.79%, this rate is lower than the figure that has been reported for allo-HCT in the recent systematic review (22).Nearly the same as our study findings, a research headed by EBMT indicated that at the time of auto-SCT, an overall survival rate of RS patients was 59% (13). However, the relapse rate in the mentioned study was 65% compared to the pooled rate of 40.23% reported in our meta-analysis study (13). These findings highlight the relatively restricted efficacy of auto-SCT in eliminating the deterioration rate of someone's state of health in RS patients (13). Furthermore, the efficacy of hematopoietic stem cell transplantation has been well proven in chronic lymphocytic leukemia, hence, the three-year survival rate in patients exceeded 50% (23-26). 

These findings are in line with EBMT study which reported a three-year survival rate of 41% in patients who received a transplant in complete remission or partial response (13). Regarding the overall survival rate, a supposedly higher rate in cases undergoing auto-SCT (versus patients with allo-SCT) might indicate the fact that a considerably higher percentage of cases had an evident sign of progressive disease at the time of allografting (13). Additionally, patients undergoing allo-SCT had been heavily pretreated and received multiple treatment regimens and lines of therapy which might negatively affect their recovery process and survival rate (13). On the other hand, allo-SCT in patients with CLL has been demonstrated to avoid the adverse effects of deletion 17p, unmutated immunoglobulin heavy-chain variable region gene (IGHV), and Zeta-chain-associated protein kinase 70 (ZAP-70) among others (24, 27-30). Another advantage for allo-SCT over auto-SCT in RS patients is that the former therapy leads to durable remissions while auto-SCT was not successful enough to show a long-lasting remission or optimal survival rate as an integrated strategy with chemotherapy or chemo immunotherapy (31-35). In fact, auto-SCT is less effective in patients with chemo refractory DLBCL, thus it cannot be mentioned as a standard therapy in DLBCL (36). On the other hand, it appears that allo-SCT can lead to a long-term disease control in almost 40% of DLBCL patients who encountered with relapse after auto-SCT (37, 38). In case of de novo diffuse large B-cell lymphomas (DLBCL), study results also introduced allo-SCT as a practical therapeutic alternative for patients who relapsed after an auto-SCT (11). Indeed, generalizing these findings for patients with RS should be considered and interpreted with more caution. Two different therapeutic mechanisms might influence the efficacy of stem cell transplantation in DLBCL-type RS. First, the dose intensity in delivering cytotoxic therapy and, second, the graft-versus-tumor activity in the case of allogeneic SCT. The assumption that graft-versus-tumor effects can eradicate malignant disorders resulted in the development of reduced intensity conditioning (RIC) regimens and consequently introduced allo-SCT as an available therapeutic strategy for elderly and cases with poor health conditions (13). Accordingly, evidence has affirmed that patients who receive allo-SCT with a reduced-intensity regimen have the longest survival (13). 

Furthermore, patients with a chemotherapy-sensitive disease who undergo SCT experience a higher rate of survival compared to cases with progressive disease (13). Therefore, both auto-SCT and reduced-intensity allo-SCT can be effective in RS patients provided that they receive transplantation procedure with chemo sensitive disease. Regarding patients with RS, Tsimberidou et al. found that similar to non-RS diffuse large B-cell lymphomas, lack of complete remission before allo-SCT led to a significantly lower survival rate compared with cases allografted in complete remission or partial response. This result proposes that before allo-SCT, the chemo sensitivity should be clarified as an important requirement for RS patients (39). 

Despite the relative efficacy that was proven regarding these treatment procedures, RS patients need an adequately durable diminution of the disease severity to get ready for auto-SCT. Accordingly, a study conducted by Parikh et al. revealed that over the last three decades only 11% of patients were able to experience the auto-SCT. This group of patients was on average of 10 years younger than other cases who did not meet the conditions of this hematopoietic cell transplantation (40). Study results added that an average survival rate for patients undergoing either auto or allo-SCT was higher compared with those who were not treated with these procedures (40). In a study by Maddocks et al., patients with RS progression after ibrutinib reported a low average survival of 3.5 months, while a study headed by EBMT revealed that relapse-free survival after allo-SCT and auto-SCT was 27% and 45% respectively. These results highlight the fact that disease progression is the main reason for discontinuing ibrutinib in 18% to 58% of patients and emphasize on the necessity for early identification and timely referral of these cases to transplant centers (20). Thus, despite the advantages of SCT therapeutic methods in terms of increasing survival rate, their limited use among RS patients for reasons such as the rapidly growing disease or the existence of underlying diseases in older adults has questioned the applicability of these novel approaches (41). 

In conclusion, due to limitations associated with the scarcity of data, it is challenging to recommend an optimal treatment approach for DLBCL-type RS patients. However, based on the available scientific data mostly derived from retrospective studies, it has been recommended that if chronic lymphocytic leukemia and DLBCL are clonally unrelated due to dissimilar immunoglobulin gene rearrangements, the disease should be treated as a de novo DLBCL; but in clonally-related cases, de novo DLBCL may result in poor clinical outcome and transformation can be a proper strategy following a clinical trial. Otherwise, chemo immunotherapy followed by reduced-intensity allo-SCT or auto-SCT has been suggested.
